# Mutations in *CERKL* and *RP1* cause retinitis pigmentosa in Pakistani families

**DOI:** 10.1038/s41439-020-0100-8

**Published:** 2020-05-12

**Authors:** Raheela Nadeem, Firoz Kabir, Jiali Li, Libe Gradstein, Xiaodong Jiao, Bushra Rauf, Muhammad Asif Naeem, Muhammad Zaman Assir, Sheikh Riazuddin, Radha Ayyagari, J. Fielding Hejtmancik, S. Amer Riazuddin

**Affiliations:** 1 National Centre of Excellence in Molecular Biology, University of the Punjab, Lahore, 53700 Pakistan; 2 The Wilmer Eye Institute, Johns Hopkins University School of Medicine, Baltimore, MD 21287 USA; 3 Ophthalmic Genetics and Visual Function Branch, National Eye Institute, National Institutes of Health, Bethesda, MD 20892 USA; 4 Department of Ophthalmology, Zhujiang Hospital, Southern Medical University, Guangzhou, 515282 China; 5 Allama Iqbal Medical College, University of Health Sciences, Lahore, 54550 Pakistan; 6 Shiley Eye Institute, University of California San Diego, La Jolla, CA 92093 USA

**Keywords:** Genetic linkage study, Genetic markers

## Abstract

This study was conducted to identify the genetic basis of retinal dystrophies in consanguineous Pakistani families. We recruited two families with retinitis pigmentosa (RP) displaying visual difficulties, including nyctalopia and constricted visual fields. Linkage analysis and Sanger sequencing resulted in the identification of a previously reported nonsense mutation, c.847C > T, in exon 5 of *CERKL* in one family and a novel four-base pair deletion in exon 4 of *RP1*, c.delAGAA4218_4221, leading to premature protein termination in the second family. Here, we report two RP-causing mutations extending the genetic heterogeneity of the disease.

## Introduction

Retinal dystrophy (RD) is a group of rare hereditary disorders that causes degeneration of the retina of the eye. RD involves progressive degeneration of retinal photoreceptors: rods, cones, or both. Among retinal dystrophies, retinitis pigmentosa (RP) primarily affects rod photoreceptors and has the highest worldwide prevalence of 1 in 4000 individuals.^1^ Loss of rod photoreceptors results in night blindness, followed by loss of peripheral vision, leading to tunnel vision. During the later stages of the disease, cone photoreceptors are compromised, resulting in reduced visual acuity and eventually complete blindness.^1,2^ RP can manifest in an autosomal recessive, autosomal dominant or X-linked pattern, while sporadic cases have also been reported. Mutations associated with autosomal recessive RP (arRP) have been identified in multiple genes (https://sph.uth.edu/retnet/home.htm).

The two multigenerational familial cases, PKRP373 and PKRP388, reported in this study were recruited from the Punjab province of Pakistan (Supplementary Figs. 1, 2). The Institutional Review Boards (IRBs) of Johns Hopkins University School of Medicine (Baltimore, MD, USA), the National Eye Institute (Bethesda, MD, USA) and the National Centre of Excellence in Molecular Biology (Lahore, Pakistan) approved this study. All participating subjects gave informed consent consistent with the tenets of the Declaration of Helsinki. Medical history was obtained by interviewing senior family members and evaluating the clinical records from previous ophthalmological examinations. Funduscopic examination was completed using a handheld Optomed Smartscope (Oulu, Finland), while the electroretinography (ERG) measurements were recorded with LKC equipment (Gaithersburg, MD, USA).

**Fig. 1:**
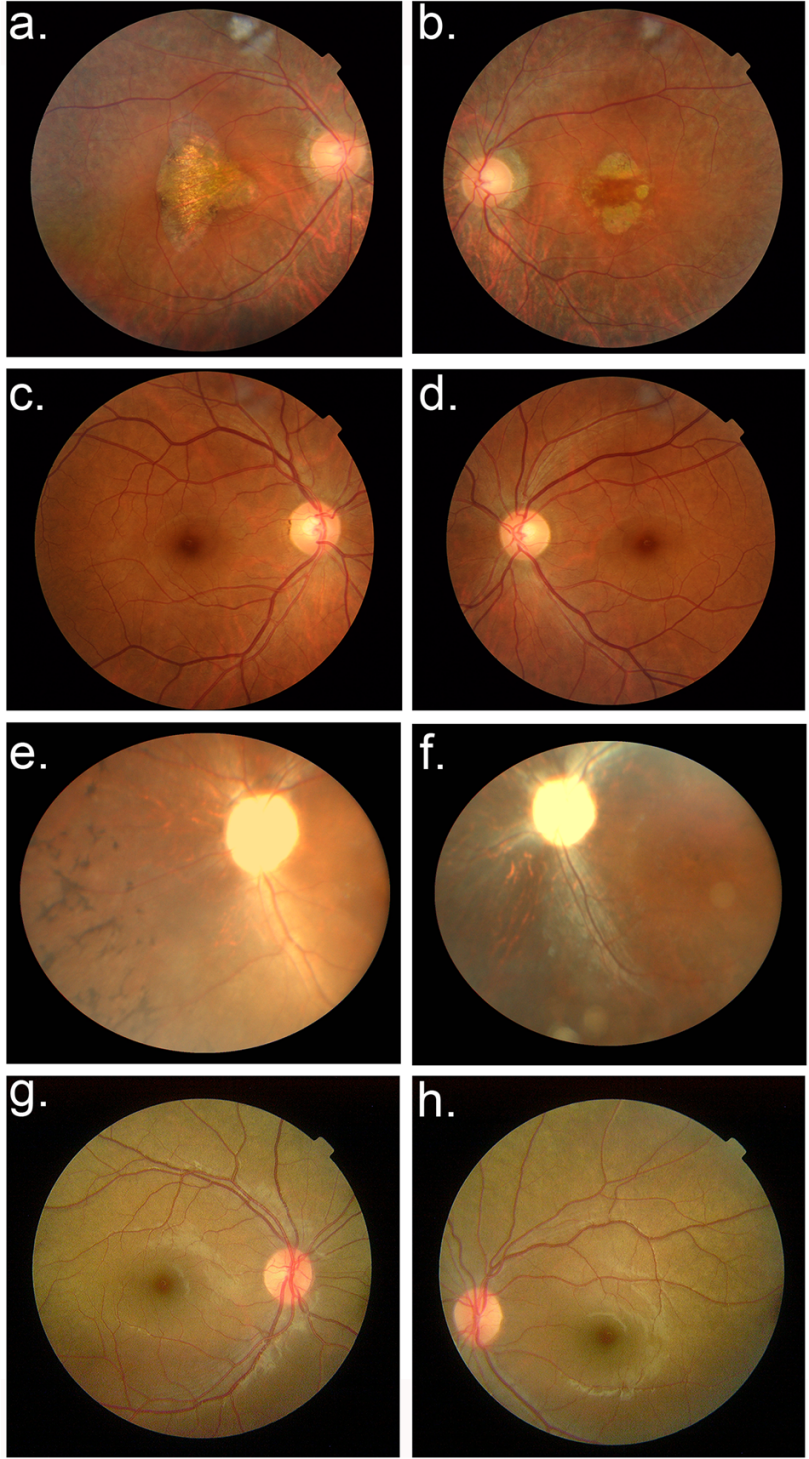
Fundus photographs of individuals examined for retinitis pigmentosa. **a**, **b** Fundus photographs (OD and OS, respectively) of individual 8 from family PKRP373 show attenuated arterioles and some optic disk pallor, as well as pronounced atrophy of the central retina (the macula) and atrophy around the optic disc. **c**, **d** The normally appearing fundus of unaffected individual 7 from family PKRP373 (OD and OS, respectively) including the macula and optic disc. **e**, **f** Fundus photographs of individual 10 from family PKRP388 (OD and OS, respectively) demonstrate vascular attenuation, marked optic disc pallor, bone-spicule pigmentation, and degenerative changes in the peripheral and central retina. **g**, **h**) The normal fundus of unaffected individual 12 from family PKRP388 (OD and OS, respectively) showing a normal caliber of retinal vessels, no signs of retinal pigmentation, and a normally appearing optic disc. *OD* Oculus Dextrus (right eye); *OS* Oculus Sinister (left eye).

**Fig. 2:**
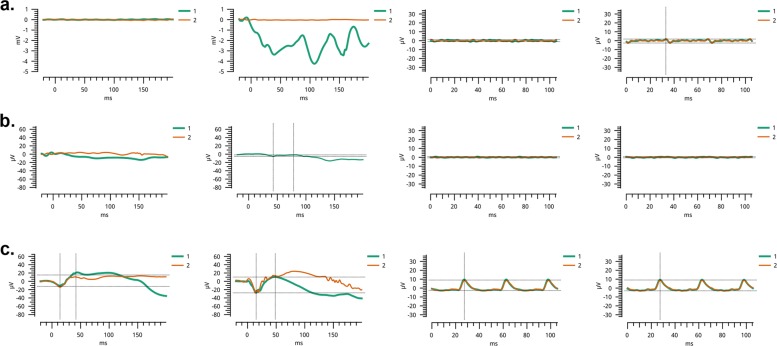
Electroretinography recordings of individuals examined for retinitis pigmentosa. Stimulus conditions: scotopic 0 dB bright flashes elicit rod responses, and photopic 0 dB and 30 Hz flickers elicit cone responses. For each individual, recording traces correspond to the following (from left to right): scotopic response OD, scotopic response OS, photopic response OD, and photopic response OS; two recordings from each eye were obtained under most conditions (1 and 2, green, and red traces, respectively). **a** Affected individual 8 of family PKRP373 exhibited nondetectable electroretinography responses (trace 1 on the second from the left plate is an artifact). **b** Affected individual 7 of family PKRP388 also showed extinguished responses under all conditions. **c** Normal responses were seen in an ethnically matched, unaffected individual. *OD* Oculus Dextrus (right eye); *OS* Oculus Sinister (left eye).

Family PKRP373 was enrolled with three affected individuals suffering from night blindness since early childhood, along with reduced visual acuity and loss of the peripheral visual field. Ophthalmic examination revealed severe degeneration of the peripheral and central retina, including the macula (the area in the central retina responsible for sharp vision), along with atrophy around the optic disc (Fig. 1a, b). The full-field ERG record illustrates a profound degree of rod and cone photoreceptor dysfunction (Fig. 2a). Likewise, during the clinical evaluation of family PKRP388, classical features of RP were evident on fundus photographs of affected members, showing bone-spicule pigment deposition, optic disc pallor and attenuated blood vessels in the retina (Fig. 1e, f). Scotopic and photopic ERGs show no response, indicating severely compromised photoreceptors (Fig. 2b). The unaffected individuals in both families had normal fundus and ERG responses (Figs. 1c, d, g, h, 2c).

Peripheral blood (~10 ml) was drawn from all participating family members, and the samples were stored in 50 ml Sterilin Falcon tubes (Sarstedt, Inc. Newton, NC, USA) with 20 mM EDTA. Genomic DNA was extracted from peripheral blood (white blood cells) by a modified nonorganic procedure as described previously^3^. An exclusion analysis for the reported autosomal recessive RP (arRP) loci and genes was completed using closely spaced short tandem repeat (STR) markers. Polymerase chain reaction (PCR) was completed in a 5 μl reaction volume as described previously^3^. A loading cocktail containing HD-400 size standards (Applied Biosystems, Foster City, CA USA) was mixed with PCR products. The resulting PCR products were resolved with an ABI 3100 Genetic Analyzer (Applied Biosystems), and genotypes were assigned with the GeneMapper software (Applied Biosystems). Two-point linkage analysis was completed with alleles obtained through the exclusion analysis using the FASTLINK version of MLINK from the LINKAGE Program Package (provided in the public domain by the Human Genome Mapping Project Resources Centre, Cambridge, UK)^4,5^.

Exclusion analysis linked the RP phenotype in family PKRP373 to chromosome 2q31.1 (Supplementary Fig. 1) with maximum two-point LOD scores of 1.12, 2.19, and 2.90 obtained with STR markers D2S384, D2S2387 and D2S2246, respectively (Supplementary Table 1). In parallel, the disease in family PKRP388 with seven affected individuals was found to be linked to chromosome 8q12.1 (Supplementary Fig. 2), with maximum two-point LOD scores of 3.22, 3.22, 3.22, and 3.20 obtained with markers D8S1110, D8S1737, D8S509, and D8S2332, respectively (Supplementary Table 2). No evidence of suggestive linkage was noted with the other previously reported RP loci and/or genes.

The linkage intervals on chromosomes 2q31.1 and 8q12.1 harbor *CERKL* and *RP1*, respectively, genes previously shown to be associated with RP. Bidirectional Sanger sequencing of *CERKL* for members of family PKRP373 identified a homozygous C > T substitution, c.847C > T, in exon 5 resulting in truncation of the protein (p.Arg283*) (Supplementary Fig. 3a–c). Likewise, the bidirectional sequencing of *RP1* identified a novel homozygous four-base pair deletion in exon 4 at position c.delAGAA4218_4221 (Supplementary Fig. 3d–f) resulting in a premature termination codon after 10 amino acids (p.E1407Qfs*10). Both mutations segregated with the disease phenotype in an autosomal recessive inheritance pattern in their respective families and were absent in 96 ethnically matched controls.

*CERKL* was first recognized to be associated with RD by a Spanish group who identified the c.847C > T (p.Arg283Ter) mutation (reported as c.769C > Tp.Arg257Ter)^6^. To date, only one pathogenic mutation in *CERKL*, c.316C > A (p.Arg106Ser) has been documented in the Pakistani population^7^. Several studies of Yemenite Jewish, Finnish, and Spanish populations have identified mutations in CERKL as a cause of RD^6,8–10^. Importantly, the affected individuals in family PKRP373 and affected individuals described in previously published reports of *CERKL*-related RD suffer from early and severe involvement of the central retina in addition to peripheral retinal degeneration^6,8–10^.

The mutation identified in family PKRP388 resides in exon 4 of *RP1* and is predicted to result in a truncated protein (losing nearly one-third of the RP1 protein). The majority of mutations that have been identified in the *RP1* cluster in exon 4, the largest (and the terminal) exon of the gene^11^. *RP1* is associated with autosomal dominant and autosomal recessive RP, as well as autosomal recessive cone-rod dystrophy and autosomal recessive macular degeneration^12^. Mutations in exon 4 display divergent clinical consequences, which indicate that genetic testing alone is insufficient for counseling, and it is important to consider the clinical findings and family history.

In conclusion, we report two causal mutations in consanguineous RP families recruited from Pakistan. The delineation of mutations in *CERKL* and *RP1* will lead to a better understanding of the physiology and underlying pathology of the disease and improve genetic counseling for the families at risk.

## Supplementary information

Supplementary Materials
