# Supplementary material for: Mutations in CERKL and RP1 cause retinitis pigmentosa in Pakistani families
Source: Hum Genome Var. 2020 May 12;7:14. doi: 10.1038/s41439-020-0100-8 (PMC7217820; doi:10.1038/s41439-020-0100-8)
Supplement: Supplementary file 1 — Supplementary Materials [file 41439_2020_100_MOESM1_ESM.pdf]

Supplementary Material

Mutations in *CERKL* and *RP1* cause retinitis pigmentosa in Pakistani families

Supplementary Material

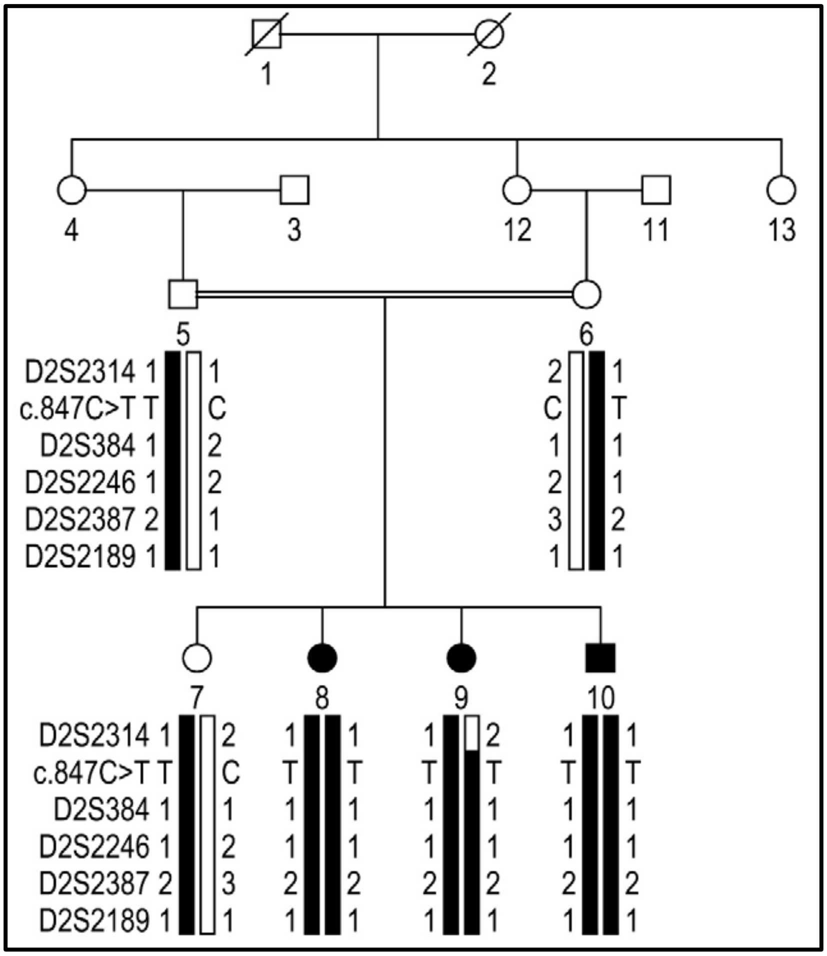

Supplementary Figure 1: Pedigree of family PKRP373 with the haplotypes of alleles on chromosome 2q31.1 and segregation of c.847C>T variation in *CERKL*. Alleles forming the risk haplotype are shaded black and alleles not co-segregating with RP are shown in white. Square: male; circle: female; filled symbol: affected individual; the double line between individuals: consanguineous marriage; diagonal line through a symbol: deceased individual.

Supplementary Material

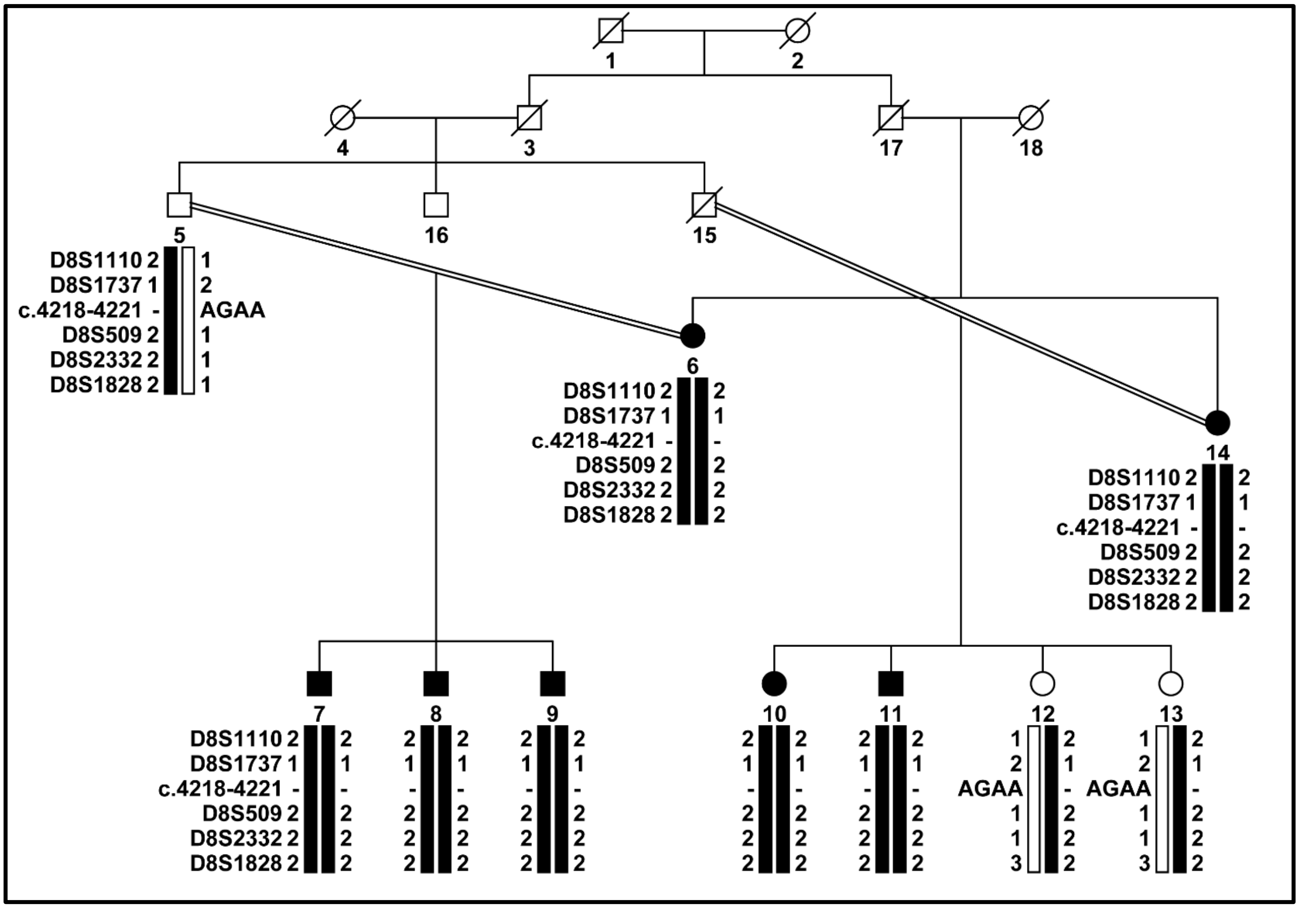

Supplementary Figure 2: Pedigree of family PKRP388 with the haplotypes of alleles on chromosome 8q12.1 and segregation of c.delAGAA4218_4221 in *RP1*. Alleles forming the risk haplotype are shaded black and alleles not co-segregating with RP are shown in white. Symbols are as described in supplementary figure 1.

Supplementary Material

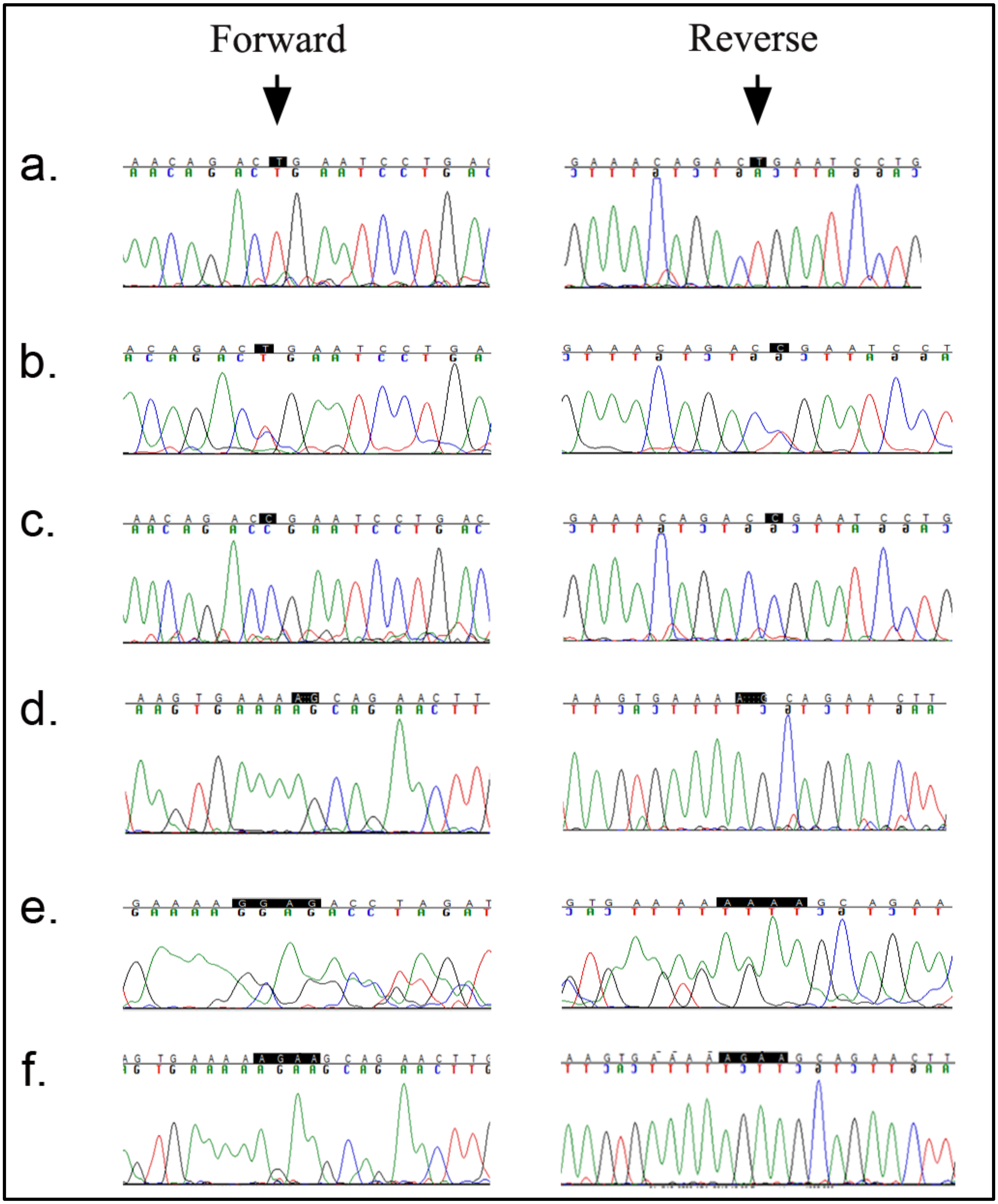

Supplementary Figure 3: Sequence chromatograms of mutant alleles identified in PKRP373 and PKRP388 responsible for retinitis pigmentosa. **a)** Affected, individual 10 of PKRP373, homozygous for the c.847C>T allele in *CERKL*, **b)** unaffected, individual 7 heterozygous carrier of the c.847C>T allele in *CERKL* and **c)** unaffected control homozygous for the wild type allele. **d)** Affected,

individual 10 of PKRP388, homozygous for the four base-pair deletion (c.delAGAA4218_4221) in *RP1*, **e)** unaffected, individual 12 of PKRP388, heterozygous for the four base-pair deletion (c.delAGAA4218_4221) in *RP1* and **f)** unaffected control homozygous for wild type allele.

Supplementary Material

Markers	cM	Mb	0.00	0.01	0.03	0.05	0.07	0.09	0.10	0.20	0.30	Zmax	θmax
**D2S2314**	182.24	175.99	− ∞	-0.60	-0.18	-0.01	0.08	0.14	0.16	0.20	0.14	0.20	0.20
**D2S384**	185.13	180.63	1.12	1.10	1.05	1.00	0.95	0.90	0.88	0.62	0.38	1.12	0.00
**D2S2246**	190.00	191.51	2.90	2.85	2.76	2.65	2.47	2.32	2.20	1.65	1.09	2.90	0.00
**D2S2387**	196.24	196.31	2.19	2.14	2.05	1.96	1.86	1.77	1.72	1.24	0.77	2.19	0.00
**D2S2189**	199.18	204.11	0.49	0.47	0.44	0.41	0.38	0.35	0.33	0.20	0.09	0.49	0.00

Supplementary Table 1: Two-point LOD scores of PKRP373 localized to chromosome 2q31.1

Supplementary Material

Markers	cM	Mb	0.00	0.01	0.03	0.05	0.07	0.09	0.10	0.20	0.30	Zmax	θmax
**D8S1110**	65.47	52.26	3.22	3.16	3.04	2.91	2.78	2.65	2.59	1.92	1.23	3.22	0.00
**D8S1737**	67.27	53.87	3.22	3.16	3.04	2.91	2.78	2.65	2.59	1.92	1.23	3.22	0.00
**D8S509**	69.40	54.68	3.22	3.16	3.04	2.91	2.78	2.65	2.59	1.92	1.23	3.22	0.00
**D8S2332**	69.40	55.21	3.20	3.14	3.01	2.89	2.76	2.63	2.56	1.89	1.21	3.19	0.00
**D8S1828**	71.00	52.26	$-\infty$	1.14	1.50	1.61	1.63	1.62	1.61	1.29	0.84	1.63	0.070

Supplementary Table 2: Two-point LOD scores of PKRP388 localized to chromosome 8q12.1
